# A new simple method for introducing an unmarked mutation into a large gene of non-competent Gram-negative bacteria by FLP/FRT recombination

**DOI:** 10.1186/1471-2180-13-86

**Published:** 2013-04-17

**Authors:** Masahito Ishikawa, Katsutoshi Hori

**Affiliations:** 1 Department of Biotechnology, Graduate School of Engineering, Nagoya University, Furo-cho, Chikusa-ku, Nagoya, 464-8603, Japan

**Keywords:** Gene disruption, FLP/FRT recombination, Non-competent bacteria, Unmarked mutation, Homologous recombination, Large gene, Gram-negative bacteria

## Abstract

**Background:**

For the disruption of a target gene in molecular microbiology, unmarked mutagenesis is preferable to marked mutagenesis because the former method raises no concern about the polar effect and leaves no selection marker. In contrast to naturally competent bacteria, there is no useful method for introducing an unmarked mutation into a large gene of non-competent bacteria. Nevertheless, large genes encoding huge proteins exist in diverse bacteria and are interesting and important for physiology and potential applications. Here we present a new method for introducing an unmarked mutation into such large genes of non-competent Gram-negative bacteria.

**Results:**

Two gene replacement plasmids, pJQFRT and pKFRT/FLP, were constructed to apply the FLP/FRT recombination system to introduce an unmarked mutation into a large gene of non-competent Gram-negative bacteria. In our methodology, pJQFRT and pKFRT/FLP are integrated into the upstream and the downstream regions of a target gene, respectively, through homologous recombination. The resultant mutant has antibiotic resistance markers, the *sacB* counter-selection marker, *flp* recombinase under the control of the *tetR* regulator, and identical FRT sites sandwiching the target gene and the markers on its chromosome. By inducing the expression of *flp* recombinase, the target gene is completely deleted together with the other genes derived from the integrated plasmids, resulting in the generation of an unmarked mutation. By this method, we constructed an unmarked mutant of *ataA*, which encodes the huge trimeric autotransporter adhesin (3,630 aa), in a non-competent Gram-negative bacterium, *Acinetobacter* sp. Tol 5. The unmarked *ataA* mutant showed the same growth rate as wild type Tol 5, but lost the adhesive properties of Tol 5, similar to the transposon-inserted mutant of *ataA* that we generated previously.

**Conclusions:**

The feasibility of our methodology was evidenced by the construction of an unmarked *ataA* mutant in the Tol 5 strain. Since FLP/FRT recombination can excise a long region of DNA exceeding 100 kb, our method has the potential to selectively disrupt much larger genes or longer regions of gene clusters than *ataA*. Our methodology allows the straightforward and efficient introduction of an unmarked mutation into a large gene or gene cluster of non-enterobacterial Gram-negative bacteria.

## Background

Disruption of a target gene is essential for revealing the functions of the gene and/or its product exhibiting an organism’s phenotype, and this process is equally applicable to microbes. The approaches used to disrupt a target gene can be divided into marked and unmarked mutation methods. The marked method requires the integration of a selectable marker, such as an antibiotic resistance gene, into a target gene. Although the marker-inserted gene becomes inactive, the marker frequently affects the expression of other genes, the so-called polar effect. In addition, marked mutants usually obtain antibiotic resistance, making it difficult to introduce an additional mutation. In contrast, the unmarked method, which is also called a null or in-frame mutation, requires deletion of the open reading frame of a target gene from the microbial chromosome, raises no concern about the polar effect, and leaves no antibiotic resistance that would prevent the introduction of an additional mutation. Therefore, the unmarked method is preferable for gene disruption.

Some bacteria can be mutated by a PCR-based method, in which a PCR product of an allele containing a marker is introduced directly into the cell and exchanged for a target gene by homologous recombination, and the marker is subsequently excised in some way when in need of an unmarked mutant [1-3]. However, this method can only be used for limited kinds of bacteria because it requires bacterial cells to be sufficiently competent to ensure uptake of linear DNA. Non-competent Gram-negative bacteria are frequently mutated by a plasmid-based method, in which plasmid DNA is introduced into the cell by bacterial conjugation [4], and allelic marker exchange is then carried out by homologous recombination between the chromosomal DNA and the introduced allele on a gene replacement plasmid [5-7]. Since single crossover mutants are dominantly obtained in the plasmid-based method, counter-selection markers such as *sacB*[8], *rpsL*[9], and mutated *pheS*[10], which confer sensitivity to sucrose, streptomycin, and *p*-chloro-phenylalanine, respectively, are used frequently to further screen double crossover mutants, especially for an unmarked mutation. However, this method is empirically ineffective for deleting large genes from the chromosome. Thus, it is difficult to characterize the function of a large gene in non-competent bacteria by using an unmarked mutation. Nevertheless, bacteria have large genes that are interesting and important for physiology and potential applications, such as cell surface proteins that have repetitive structures and are involved in cell adhesion and biofilm formation [11-15]. The repeats of a gene also disturb recombination at the targeted site on the chromosome and complicate the introduction of an unmarked mutation. Since there is no effective method for introducing an unmarked mutation that targets such large genes in non-competent bacteria, marked mutants have been used to characterize their functions.

The site-specific recombinase FLP, which is a yeast protein, works efficiently in a variety of prokaryotic and eukaryotic hosts [1,2,5,16,17]. When FLP recognition target (FRT) sites are aligned on the chromosome of a host cell in the same direction, FLP recombinase binds to them and specifically excises the region sandwiched between the two FRT sites. In both the PCR-based and the plasmid-based unmarked methods, the FLP/FRT recombination system has been employed to eliminate selectable markers inserted into the chromosome [1,2,5,18].

*Acinetobacter* sp. Tol 5 is an interesting Gram-negative bacterium that can metabolize various kinds of chemicals, including aromatic hydrocarbons, ethanol, triacylglycerol, and lactate [19,20], has a hydrophobic cell surface that can adsorb to oil surfaces [21,22], autoagglutinates [21,23,24], and exhibits high adhesiveness to various abiotic surfaces ranging from hydrophobic plastics to hydrophilic glass and stainless steel by bacterionanofibers [20,24-26]. AtaA is a huge protein (3,630 aa) with a multi-repetitive structure, belongs to the trimeric autotransporter adhesin family [27], and forms an essential nanofiber for the adhesive phenotype of Tol 5 [28]. Previously, we constructed a marked mutant of *ataA* by exchanging it with a transposon cassette-inserted allele. Since the competency of Tol 5 was quite low, allelic marker exchange was performed by the plasmid-based method using the *sacB* marker. Although an unmarked mutant is more preferable to a marked mutant, the excision of *ataA* from the chromosome of Tol 5 was considered quite difficult due to the size and the repetitive structure of *ataA* (10,893 bp). In this study, we focused on the ability of FLP/FRT recombination to excise a long region of chromosomal DNA [29] and considered it to be suitable for introducing an unmarked mutation into a large gene. Here, we developed a new system for targeted gene disruption by FLP/FRT recombination in non-competent Gram-negative bacteria, and then constructed an unmarked *ataA* mutant from *Acinetobacter* sp. Tol 5 in order to demonstrate the feasibility of our methodology.

## Results and discussion

### A new unmarked plasmid-based mutation for non-competent bacteria

**Figure 1:**
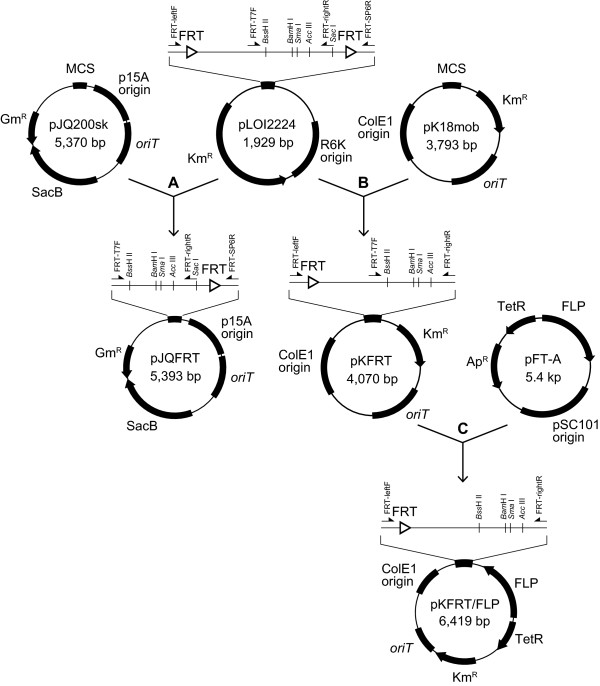
**Plasmids constructed to introduce an unmarked mutation into a large gene of non-competent bacteria.** (**A**, **B**) Multiple cloning sites (MCS) of pJQ200sk and pK18mob were substituted with that of pLOI2224, generating pJQFRT and pKFRT, respectively. The pJQFRT plasmid contains a single FRT site adjacent to a multiple cloning site; p15A origin, a replication origin of *E. coli*; *oriT*, origin of transfer; SacB, a counter-selection marker; and Gm^R^, a gentamicin resistance marker. The arrows indicate the primers used in PCR to amplify the substitute MCS. The nucleotide sequences of these primers are shown in Table 2. (**C**) A cassette containing *tetR-*P*tet* promoter and *flp* recombinase amplified by PCR from pFT-A was ligated with the inverse-PCR product of pKFRT. The resultant pKFRT/FLP plasmid contains a single FRT site adjacent to a multiple cloning site; TetR-FLP, *flp* recombinase gene under the control of the *tetR* regulation system; Km^R^, a kanamycin resistance marker; *oriT*, origin of transfer; and ColE1 origin, a replication origin of *E. coli*.

**Figure 2:**
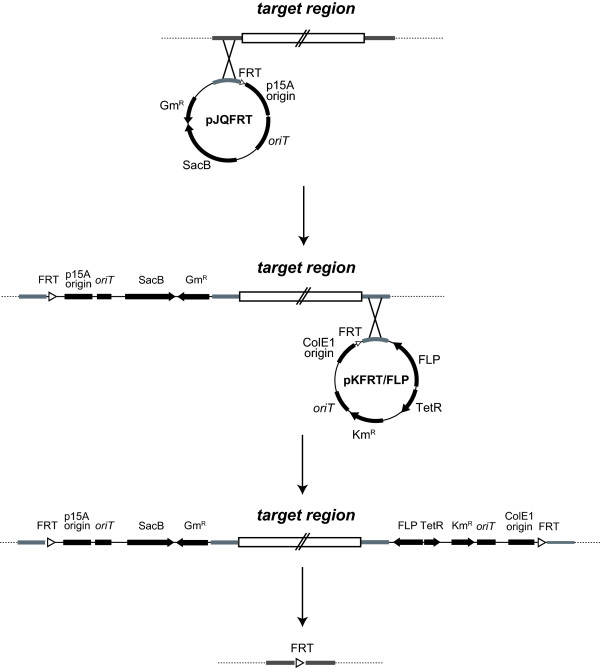
**Scheme for the unmarked deletion of a large gene by FLP/FRT recombination.** The plasmid pJQFRT with the insertion of the upstream region of the target gene is integrated into the host chromosome by homologous recombination. Next, the plasmid pKFRT/FLP with the insertion of the downstream region of the target gene is integrated into the host chromosome by homologous recombination. As a result, the target gene is sandwiched between the two integrated plasmids. The expression of *flp* is induced by adding anhydrotetracycline, and then the target region is excised together with the integrated plasmids bracketed by the two FRT sites, leaving a single FRT site.

To apply the FLP/FRT recombination system to unmarked mutagenesis, a target gene has to be sandwiched between two identical FRT sites on the chromosome. For non-competent bacteria that cannot uptake linear DNA, we developed a new plasmid-based method for unmarked mutagenesis in which the FLP/FRT recombination system can be employed. We constructed two new mobile plasmids (Figure 1): pJQFRT, which harbors the *sacB* counter-selection marker and the gentamicin resistance selection marker, and pKFRT/FLP, which harbors the kanamycin resistance selection marker and *flp* recombinase gene under the control of the *tetR* regulator. Both plasmids also harbor a single FRT site adjacent to a multiple cloning site for the insertion of a homologous region upstream or downstream of a target gene. Since these plasmids contain *oriT*, which is the origin of conjugative transfer, they can be readily introduced into a non-competent bacterium from a donor strain that possesses *tra* genes by bacterial conjugation [4]. The scheme for the unmarked deletion of a target gene using these constructed plasmids is shown in Figure 2. ColE1 and p15A replicons do not work in many Gram-negative bacteria, except for *Escherichia coli* and a limited species of *Enterobacteriaceae*. Since the introduced plasmids cannot be replicated in a non-enterobacterial cell, they are integrated into the chromosome by a single crossover event at the homologous site. When pJQFRT and pKFRT/FLP are integrated into the upstream and downstream regions of a target gene, respectively, in the resultant mutant, the original target gene is sandwiched between the sequences derived from the integrated vectors containing antibiotic resistance markers, the *sacB* marker, and *flp* recombinase under the control of the *tetR* regulator, all of which are bracketed by identical FRT sites in the same direction. In the absence of an inducer for the *tet* promoter, TetR tightly regulates the expression of *flp* recombinase, and the plasmid-integrated mutant is stable. When the expression of *flp* recombinase is induced, FLP recombinase excises the FRT bracketing sequences containing the target gene on the chromosome, resulting in the introduction of an unmarked mutation. The unmarked mutant also lacks the plasmid-derived regions, including the *tetR-flp* cassette, the origins of replication, and the selection markers, except for the single FRT site. Therefore, the generated mutant can be readily screened on an agar plate containing sucrose.

In our methodology, the new gene replacement plasmids pJQFRT and pKFRT/FLP are used for introducing the unmarked mutation. Since these plasmids are mobilized by bacterial conjugation, there is no concern about the nucleolytic degradation of the introduced plasmid DNA, unlike linear DNA. Besides, *flp* recombinase is cloned under the regulation of the *tet* promoter in pKFRT/FLP and is integrated into the chromosome of the recipient strain after homologous recombination. Therefore, our method obviates the need for helper plasmids expressing FLP recombinase and λ Red recombinase, which prevents degradation of the introduced linear DNA [30]. Our method can be used in various species of Gram-negative bacteria except for *E. coli* and some enterobacteria, independent of their competency and recombination ability.

### Implementation of the new method for the deletion of a large gene from the *Acinetobacter* sp. Tol 5 chromosome

To demonstrate the feasibility of the new mutagenesis method described above, we constructed an unmarked mutant of *ataA* in *Acinetobacter* sp. Tol 5. This is a good model for the introduction of an unmarked mutation into a large gene of non-competent Gram-negative bacteria because Tol 5 has quite low competency, even by electroporation, and *ataA* is 10,893 bp long.

**Figure 3:**
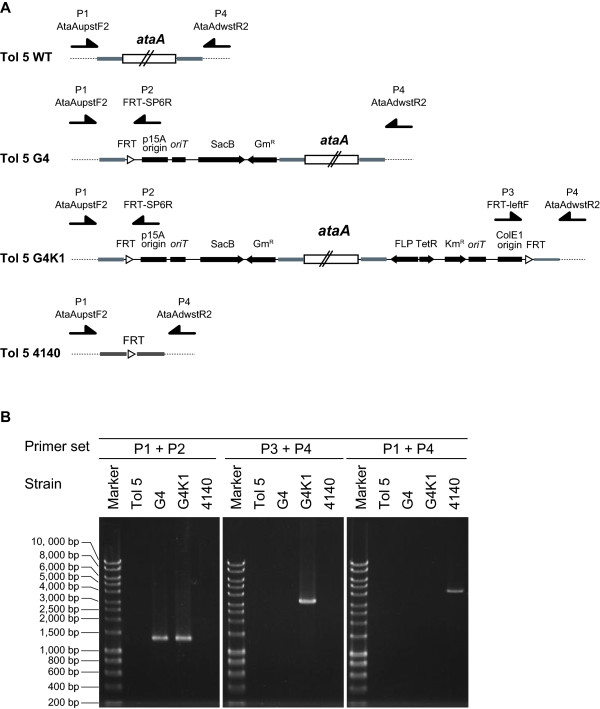
**Construction of an unmarked mutant of *ataA *from *Acinetobacter *sp. Tol 5.** (**A**) Genetic organization around *ataA* in *Acinetobacter* sp. Tol 5 and its derivative mutants obtained by plasmid integration and FLP/FRT recombination. The arrows indicate the primers used in PCR analysis for the confirmation of the constructs. (**B**) PCR confirmation of plasmid integration and the deletion of *ataA* in the Tol 5 derivatives. Chromosomal DNA was extracted as a template for PCR from Tol 5 and its derivatives (G4, G4K1, and 4140). PCR analyses were performed by using three different primer sets: P1 (AtaAupstF2) + P2 (FRT-SP6R), P3 (FRT-leftF) + P4 (AtaAdwstR2), and P1 + P4. The nucleotide sequences of these primers are shown in Table 2.

To insert the FRT sites into the upstream and downstream regions of *ataA*, a 1.0-kb DNA fragment containing the upstream region of the start codon of *ataA* was amplified by PCR using the primers AtaAupstF/AtaAupstR and inserted into pJQFRT at the *Bam*HI site, generating pJQFRT_AtaAupstream. Another 2.8-kb DNA fragment containing the downstream region of the stop codon of *ataA* was also amplified by PCR using the primers AtaAdwstF/AtaAdwstR and inserted into pKFRT/FLP at the *Bam*HI site, generating pKFRT/FLP_AtaAdownstream. The plasmid pJQFRT_AtaAupstream was transferred into Tol 5 cells from the donor *E. coli* strain through conjugation, and integrated into the chromosome of Tol 5 by homologous recombination. The plasmid-integrated mutant of Tol 5 (Tol 5 G4) was selected on an agar plate containing gentamicin. Subsequently, the plasmid pKFRT/FLP_AtaAdownstream was transferred into Tol 5 G4 cells from the donor, and integrated into the chromosome of Tol 5 G4. The mutant that has the chromosome integrated by the two plasmids (Tol 5 G4K1) was selected on an agar plate containing kanamycin and gentamicin. Integration of the plasmids was also confirmed by PCR using two primer sets, AtaAupstF2/FRT-SP6R and FRT-leftF/AtaAdwstR2 (Figure 3). The PCR amplicons were detected in Tol 5 G4 and G4K1, but not in Tol 5, indicating the correct insertion of the plasmids into the chromosome of Tol 5.

To excise *ataA* together with the region derived from the integrated plasmids, *flp* recombinase was induced by adding anhydrotetracycline to the culture of Tol 5 G4K1. After incubation for recombination by FLP, the cell suspension was plated on a medium containing 5% sucrose. Although unmarked *ataA* mutants were selectable on the sucrose plate, the sucrose-resistant colonies possibly included spontaneous *sacB* mutants. Thus, we confirmed the excision of *ataA* by PCR using the primers AtaAupstF2/AtaAdwstR2, which anneal to the outside of the flanking regions of *ataA* used as the homologous sites for recombination (Figure 3). PCR amplicons were not detected from template chromosomes of Tol 5, G4, and G4K1 due to the large size of *ataA*. In contrast, a small DNA fragment was amplified from the chromosome of a sucrose-resistant mutant, Tol 5 4140, indicating the excision of *ataA*. Sequencing of the amplicon proved that *ataA* and the regions derived from the two plasmids were completely excised from the chromosome, and that sequences of the 1-kb and 2.8-kb flanking regions of *ataA* coincided with those of wild type Tol 5 (Tol 5 WT).

**Figure 4:**
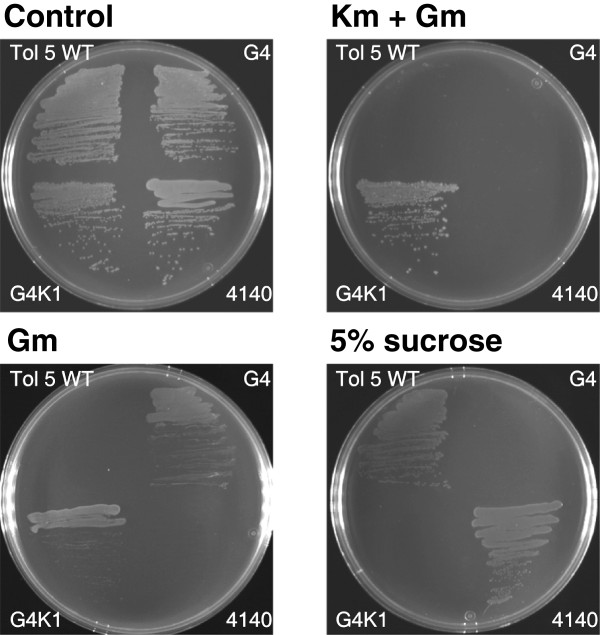
**Plating tests to confirm the presence or excision of the selection markers.** Wild type *Acinetobacter* sp. Tol 5 (Tol 5 WT), the plasmid-integrated mutants Tol 5 G4 (G4) and Tol 5 G4K1 (G4K1), and the unmarked *ataA* mutant Tol 5 4140 (4140) were streaked on BS (Control), BS containing 100 μg/ml gentamicin (Gm), BS containing 100 μg/ml gentamicin and 100 μg/ml kanamycin (Gm + Km), and BS containing 5% sucrose (5% sucrose) plates, and incubated with a supply of toluene as a carbon source.

Plating tests also showed that the respective mutants obtained in the procedure for the unmarked mutagenesis of Tol 5 exhibited the expected resistance/susceptibility against antibiotics and sucrose (Figure 4). The plasmid-integrated mutants G4 and G4K1 showed resistance to only gentamicin and to both gentamicin and kanamycin, respectively, but both strains were not viable on a plate containing 5% sucrose. In contrast, the unmarked *ataA* mutant Tol 5 4140 grew on the sucrose plate, but was sensitive to gentamicin and kanamycin, like Tol 5 WT, indicating that the marker genes did not remain in Tol 5 4140 cells.

**Figure 5:**
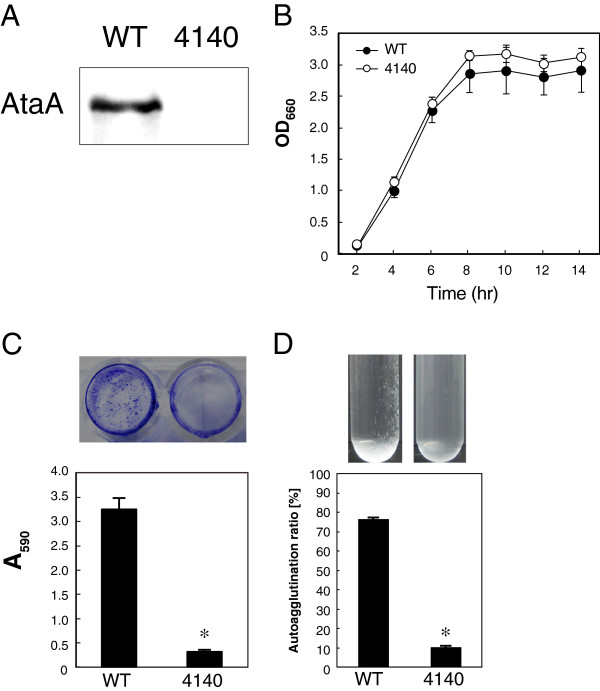
**Examination of the phenotype of the unmarked mutant Tol 5 4140.** (**A**) Immunodetection of AtaA using an anti-AtaA antiserum against whole cell lysates prepared from Tol 5 WT and the 4140 mutant. (**B**) Growth curve of Tol 5 WT and the 4140 mutant in LB medium at 28°C, with shaking at 115 rpm. Data are expressed as the mean and SD obtained from 3 independent cultures. (**C**) Adhesion of Tol 5 WT and the 4140 mutant to a polystyrene surface. The photograph indicates the stained cells adhering to a 48-well plate. Data are expressed as the mean and SEM (n = 3). Statistical significance, *P < 0.01. (**D**) Autoagglutination assay of Tol 5 WT and the 4140 mutant by the tube-settling assay. The photographs indicate test tubes after a 3-h incubation without agitation. Data are expressed as the mean and SEM (n = 3). Statistical significance, *P < 0.001.

Immunodetection using anti-AtaA antibody proved the lack of *ataA* expression in Tol 5 4140 (Figure 5A). We also confirmed that the growth rate of Tol 5 4140 was equal to that of Tol 5 WT, suggesting no effect of the unmarked *ataA* mutation on other genes that affect cell growth (Figure 5B). Previously, we reported that AtaA is an essential protein for the autoagglutinating nature and high adhesiveness of Tol 5 cells [28]. To characterize the adhesive properties of Tol 5 4140, we performed adherence and autoagglutination assays, as described previously [24,28]. As a result, Tol 5 4140 was shown to have lost the high adhesiveness of Tol 5 WT cells to a polystyrene surface (Figure 5C). In the autoagglutination assay by the tube-settling method, Tol 5 4140 cells were dispersed and the cell suspension remained cloudy even after a 3-h incubation, while Tol 5 WT cells autoagglutinated and formed a sediment at the bottom of the tube, showing the significantly decreased autoagglutination ratio of Tol 5 4140 cells compared with Tol 5 WT cells (Figure 5D). Thus, the less adhesive phenotype of Tol 5 4140 was confirmed to be similar to that of a marked *ataA* mutant that we constructed previously [28]. Therefore, we successfully constructed a more preferable mutant of *ataA* using our new methodology.

Although, in this study, we constructed the unmarked *ataA* mutant by excising a 10-kb segment from the chromosome of Tol 5, our new method can theoretically be used to disrupt a larger gene in other non-competent Gram-negative bacteria because Pósfai *et al.* successfully excised 51-kb and 110-kb DNA segments from the chromosome of *E. coli* K-12 MG1655 by FLP/FRT recombination [29]. Bap (8,620 aa) from *Acinetobacter baumannii* 307–0294, LapA (8,683 aa) from *Pseudomonas fluorescens* WCS365, and LapF (6,310 aa) from *Pseudomonas putida* KT2440 are larger proteins than AtaA (3,630 aa) and play an important role in adhesion to solid surfaces and biofilm formation [11,14,15,31]. Since there was no useful method for introducing an unmarked mutation into large genes encoding them, random transposon mutants have been used to characterize the phenotype generated by a deficiency of those genes. By using our new unmarked method, such large genes can be easily and efficiently deleted from non-competent Gram-negative bacteria, and mutants that are more appropriate than marked mutants for the analysis of phenotypic changes can be obtained. In addition to functional analyses of large genes, our unmarked method would be effective for the metabolic engineering of bacteria to produce conventional fermentation products, biofuels, medicines, and chemicals by deleting long regions of metabolism-related gene clusters disturbing their production.

## Conclusion

We designed two gene replacement plasmids and developed a new methodology for the construction of an unmarked mutant using the FLP/FRT recombination system. This methodology overcomes the problems associated with introducing an unmarked mutation into a large gene of non-competent Gram-negative bacteria. Using this method, we successfully constructed an unmarked mutant of *ataA* of Tol 5, which is 10,893 bp long. The plasmids and the methodology should be applicable to a wide range of Gram-negative bacteria except for *E. coli* and some enterobacteria and are expected to be useful tools to characterize the functions of large genes.

## Methods

### Bacterial strains, plasmids, and growth conditions

The bacterial strains used in this study are listed in Table 1. *Acinetobacter* sp. Tol 5 and its derivative mutants were grown in basal salt (BS) medium supplemented with toluene or LB medium at 28°C, as described previously [28]. *E. coli* strains were grown in LB medium at 37°C. Antibiotics were used at the following concentrations when required: gentamicin (100 μg/ml) and kanamycin (100 μg/ml) for Tol 5 derivative mutants; gentamicin (10 μg/ml) and kanamycin (50 μg/ml) for *E. coli* strains.

**Table 1:** Bacterial strains and plasmids used in this study

**Strain**	**Description**	**Reference**
*Acinetobacter* sp.		
Tol 5	Wild type strain	[19]
G4	A Tol 5 mutant constructed by insertion of a FRT site in the upstream of *ataA* of Tol 5, Gm^r^, SacB	This study
G4K1	A Tol 5 mutant constructed by additional insertion of a FRT site in the downstream of *ataA* of G4, Gm^r^, Km^r^, SacB	This study
4140	Unmarked Δ*ataA* mutant of Tol 5 constructed by FLP/FRT recombination in G4K1	This study
*E. coli*		
DH5α	Host for routine cloning	TaKaRa
S17-1	Donor strain for conjugation	[4]
Plasmid		
pJQ200sk	Mobile plasmid, SacB, Gm^r^	[32]
pK18mob	Mobile plasmid, Km^r^	[33]
pLOI2224	Source of FRT sites, Km^r^	[34]
pFT-A	Source of FLP recombinase and *tetR*, Amp^r^	[34]
pJQFRT	Gene replacement vector harboring a single FRT sequence, SacB, and Gm^r^	This study
pKFRT	Mobile plasmid harboring a single FRT sequence, Km^r^	This study
pKFRT/FLP	Gene replacement vector harboring a single FRT sequence, FLP recombinase under the control of P*tet* promoter, and Km^r^	This study
pJQFRT_AtaAupstream	A 1.0-kb fragment containing the upstream region of *ataA* ligated into the BamHI site of pJQFRT	This study
pKFRT/FLP_AtaAdownstream	A 2.8-kb fragment containing the downstream region of *ataA* ligated into the BamHI site of pKFRT/FLP	This study

### Genetic manipulation

General DNA manipulations, such as PCR, restriction enzyme digestion, and ligation, were performed using standard protocols. The plasmids and primers used in this study are detailed in Table 1 and 2, respectively.

**Table 2:** Primers used in this study

**Primer**	**Sequence (5′ → 3′)**
FRT-leftF	AATCCATCTTGTTCAATCATGC
FRT-rightR	AATTCGAGCTCGGGAAGATC
FRT-T7F	AAATTAATACGACTCACTATAGG
FRT-SP6R	TACGATTTAGGTGACACTATAG
Inv-pUC118F	CAACGTCGTGACTGGGAAAAC
Inv-pUC118R	TCATGGTCATAGCTGTTTCCTG
TetR-FLP2F	CGATGGGTGGTTAACTCGAC
TetR-FLP2R	ACAGGACGGGTGTGGTCG
AtaAupstF	CGCGGATCCGATCTTCAAAGGTTGTGCTCAG
AtaAupstF2	AACGCAAGTTGTTTTACTGC
AtaAupstR	CGCGGATCCTAGAAGCTGTAGCAGTTGTTCC
AtaAdwstF	CGCGGATCCACTCGACAGGGAAGATCTTC
AtaAdwstR	CGCGGATCCAATTGAATCATCAACACCTGCTG
AtaAdwstR2	TACGTCGAGCAGCTAAGGTC

Underlines indicate *Bam*HI site.

### Construction of pJQFRT and pKFRT/FLP

Two mobile plasmids, pJQ200sk [32] and pK18mob [33], were used as the plasmid backbone. To remove their original multiple cloning sites, inverse-PCR was performed using the primers Inv-pUC118F/Inv-pUC118R. Substitute multiple cloning sites containing a single FRT sequence were amplified by PCR from pLOI 2224 [34] using the primer sets FRT-T7F/FRT-SP6R and FRT-leftF/FRT-rightR, and then ligated with the inverse-PCR products of pJQ200sk and pK18mob, generating pJQFRT and pKFRT, respectively. To introduce the FLP recombinase gene under the control of an inducible promoter into pKFRT, inverse-PCR was performed using the primers FRT-rightR/Inv-pUC118F. A cassette containing *tetR*, the P*tet* promoter, and *flp* recombinase was amplified by PCR from pFT-A [34] using TetR-FLP2F/TetR-FLP2R, and then ligated with the inverse-PCR product of pKFRT, generating pKFRT/FLP. The sequence data have been deposited in DDBJ/EMBL/GenBank: accession numbers [AB773261] for pJQFRT and [AB773262] for pKFRT/FLP.

### Construction of an unmarked *ataA* mutant of *Acinetobacter* sp. Tol 5

The Tol 5 strain was mated with *E. coli* S17-1 harboring pJQFRT_AtaAupstream on LB medium at 28°C for 20 h. The cells were collected in 1 ml of a 0.85% NaCl solution, plated on a BS agar plate containing gentamicin (100 μg/ml), supplied with toluene vapor as a carbon source, and incubated at 28°C for 2 days. The resulting colonies, which were resistant to gentamicin, were confirmed for the chromosomal integration of the plasmid by PCR using the primers AtaAupstF2/FRT-SP6R; thus, the Tol 5 G4 mutant was obtained. Subsequently, Tol 5 G4 was mated with *E.coli* S17-1 harboring pKFRT/FLP_AtaAdownstream using the same procedure described above, except for the use of a selection plate containing kanamycin (100 μg/ml) and gentamicin (100 μg/ml). The resulting colonies, which were resistant to gentamicin and kanamycin, were confirmed for the chromosomal integration of the plasmid by PCR using the primers FRT-leftF/AtaAdwstR2; thus, the Tol 5 G4 K1 mutant was obtained. For the excision of *ataA* and markers by FLP/FRT recombination, Tol 5 G4K1 was pre-cultured in 2 ml LB medium overnight. The overnight culture was diluted 1:100 in 20 ml fresh LB medium without antibiotics and incubated at 28°C. When the optical density of the culture broth at 660 nm reached 0.5, anhydrotetracycline was added to a final concentration of 400 ng/ml. After a 6 h incubation to induce the expression of FLP, Tol 5 G4K1 cells were seeded on a BS agar plate containing 5% sucrose and incubated at 28°C for 24 h. The resultant colonies, which were resistant to sucrose, were transferred using toothpicks to gentamicin- and kanamycin-containing BS agar plates. Desirable mutants that were sensitive to the antibiotics, but resistant to sucrose, were examined for the successful excision of the target region by PCR using the primers AtaAupstF2/AtaAdwstR2; thus, the unmarked mutant Tol 5 4140 was obtained.

### Protein manipulation

*Acinetobacter* strains were grown to the stationary phase in LB medium. The optical density (OD) at 660 nm of their cultures was adjusted to 1.0 with flesh LB medium. One milliliter of the cell suspension was harvested by centrifugation, resuspended in 50 μl of SDS-PAGE sample buffer, and boiled at 95°C for 5 min. The prepared whole cell lysates were subjected to Western-blot and immunodetection as described previously [24].

### Adherence and autoagglutination assays

*Acinetobacter* strains were grown in LB medium, harvested by centrifugation, and resuspended in BS-N medium [22] that did not contain carbon or nitrogen sources. The cell suspensions were subjected to the adherence and autoagglutination assays as described previously [24,28].

## Competing interests

The authors have declared that no competing interests exist.

## Authors’ contributions

MI performed most of experiments and wrote the manuscript. KH designed the study and wrote the manuscript. Both authors have read and approved the final manuscript.
